# A Comparable Study of CNN-Based Single Image Super-Resolution for Space-Based Imaging Sensors

**DOI:** 10.3390/s19143234

**Published:** 2019-07-23

**Authors:** Haopeng Zhang, Pengrui Wang, Cong Zhang, Zhiguo Jiang

**Affiliations:** 1 Image Processing Center, School of Astronautics, Beihang University, Beijing 102206, China; 2 Key Laboratory of Spacecraft Design Optimization and Dynamic Simulation Technologies, Ministry of Education, Beijing 102206, China; 3 Beijing Key Laboratory of Digital Media, Beijing 102206, China

**Keywords:** image super-resolution, space object, convolutional neural network, deep learning

## Abstract

In the case of space-based space surveillance (SBSS), images of the target space objects captured by space-based imaging sensors usually suffer from low spatial resolution due to the extremely long distance between the target and the imaging sensor. Image super-resolution is an effective data processing operation to get informative high resolution images. In this paper, we comparably study four recent popular models for single image super-resolution based on convolutional neural networks (CNNs) with the purpose of space applications. We specially fine-tune the super-resolution models designed for natural images using simulated images of space objects, and test the performance of different CNN-based models in different conditions that are mainly considered for SBSS. Experimental results show the advantages and drawbacks of these models, which could be helpful for the choice of proper CNN-based super-resolution method to deal with image data of space objects.

## 1. Introduction

The Space-Based Space Surveillance (SBSS) satellite [1], launched in September 2010, is a significant stepping stone towards a functional space-based space surveillance constellation. In February 2013, the Near-Earth Object Surveillance Satellite (NEOSSat) [2] was launched, which is the first space telescope dedicated to detecting and tracking asteroids and satellites. Optical imaging sensors of the vision systems aboard these satellites are the eyes for us to monitor the space. Previous researches have translated the information provided by space-based imaging sensors into many practical applications, such as autonomous rendezvous and docking [3,4,5], vision-based landing [6], position and pose estimation [7,8,9,10], space robotics and on-orbit serving [11,12,13,14], satellite recognition [15,16,17,18], 3D structure reconstruction [19,20], etc. These works have proved that high-resolution images play an important role in applications, because they contain richer information which is needed to achieve better performance in the tasks. However, it is a common scene that images of the target space objects captured by space-based imaging sensors usually suffer from low spatial resolution due to the extremely long distance between the target and the imaging sensor. Such a problem can be typically solved by image super-resolution.

The goal of image super-resolution (SR) is to restore a visually pleasing high-resolution (HR) image from a low-resolution (LR) input image or video sequence. HR images have higher pixel densities and finer details than LR images. Image SR has been proved to be of great significance in many applications, such as video surveillance [21,22,23], ultra-high definition TV [24], low-resolution face recognition [25,26,27,28,29] and remote sensing imaging [30,31]. Benefiting from its broad application prospects, SR has attracted huge interest, and currently is one of the most active research topics in image processing and computer vision. Early interpolation-based image SR methods [32,33,34] are extremely simple and fast. Unfortunately, severe aliasing and blurring effects make interpolation-based SR suboptimal in restoring fine texture details. Reconstruction-based image SR methods [35,36,37] combine elaborately designed image prior models with reconstruction constraints, and can restore fine structures. However, these image priors usually are incapable of modeling complex and varying contexts of natural images. In the past decade, most researches focus on learning-based image SR [38,39,40]. It utilizes machine learning techniques to capture the relationships between LR image patches and their HR counterparts from some samples. Recently, due to fast advances in deep learning, especially convolutional neural networks (CNNs), CNN-based SR [41,42,43,44,45] has shown promising performance in certain applications. However, there are still many challenging open topics of deep learning for image SR, e.g., new objective functions, new architectures, large scale images, depth images, various types of corruption, new applications, etc.

Therefore, this paper emphasizes the important role of CNN for single image SR with the purpose of space application. We comparably study four recently popular models including SRCNN [41] (Super-Resolution Convolutional Neural Network), FSRCNN [42] (Fast Super-Resolution Convolutional Neural Network), VDSR [44] (Very Deep Super-resolution Convolutional Networks), and DRCN [43] (Deeply-Recursive Convolutional Networks) for single image super-resolution based on CNNs. In view of the differences between natural images and images of space objects, we specially fine-tune the super-resolution models mentioned above using simulated images of space objects, and test the performance of different CNN-based models in typical conditions that are common for SBSS. Our experimental results obviously show the advantages and disadvantages of these models, thus, could be helpful for the choice of proper CNN-based super-resolution method to deal with image data of space-based sensors.

The rest of this paper is organized as follows. Section 2 describes the four CNN-based SR methods briefly and shows parameters used in this paper in detail to benefit researchers in this field. Section 3 demonstrates extensive experiments we have done to compare these four models comprehensively. Section 4 gives discussions about the experimental results. Section 5 concludes this paper.

## 2. Methods and Network Structures

### 2.1. SRCNN

**Figure 1:**
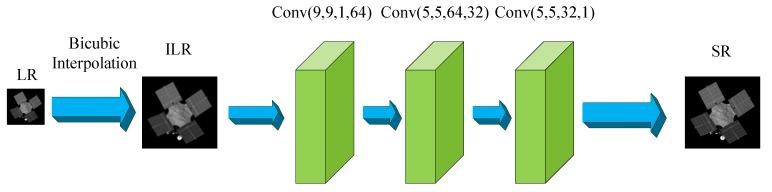
Network structure of SRCNN used in this paper. ILR, interpolated low-resolution image.

SRCNN [41] (Super-Resolution Convolutional Neural Network) is the first deep learning method for single image super-resolution, which can directly learn an end-to-end mapping between the low/high-resolution images. The network structure layout is simple as shown in Figure 1. It only contains three layers, and each layer has a convolution layer with an activation function. The input image of the network is a bicubic interpolation image of a low-resolution image, with the same size as the output HR images. The first layer mainly extracts patches and representations of low-resolution images. The second layer maps the $n_{1}-dimensional$ representations (feature vectors) of several patches into an $n_{2}-dimensional$ one, making a non-linear mapping. The number of patches for each mapping operation depends on the kernel size of the second convolution layer. Then the last layer realizes the reconstruction of high-resolution image. The parameters of SRCNN used in this paper are shown in Table 1, which are optimized to achieve the best performance of SRCNN, because of gradient vanishing, increasing the numbers of network layers cannot improve the performance of SRCNN.

### 2.2. FSRCNN

**Figure 2:**
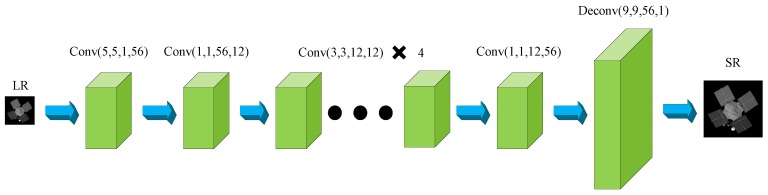
Network structure of FSRCNN used in this paper.

FSRCNN [42] (Fast Super-Resolution Convolutional Neural Network) is an upgraded version of SRCNN, focusing on accelerating the speed of high-resolution reconstruction. The structure of FSRCNN is a little more complicated and can be roughly divided into five parts, i.e., feature extraction, shrinking, mapping, expanding and deconvolution, as seen in Figure 2. The deconvolution layer is an important improvement which makes it possible to learn the mapping directly from the original low-resolution image to the high-resolution one without the interpolation operation at the beginning as SRCNN. In this way, the size of the input image does not need to be enlarged, which reduces the computation and improves the speed. As the non-linear mapping of SRCNN is operated in higher dimensional space, which is complex and time-consuming. FSRCNN solves this problem by adding a shrinking layer before the mapping operation to reduce the feature dimension. Besides, an expanding layer after the mapping layer is also added for better generating the HR image. The speed of FSRCNN is much faster than SRCNN, and the performance of FSRCNN is better as well. Table 2 shows the parameters of FSRCNN used in this paper in detail. The parameters of FSRCNN refer to the original work.

### 2.3. VDSR

**Figure 3:**
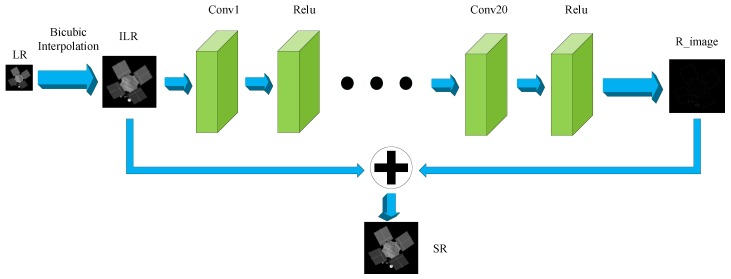
Network structure of VDSR used in this paper. ILR, interpolated low-resolution image; R_image, residual image.

VDSR [44] (Very Deep Super-resolution Convolutional Networks) explores the improvement of SR performance with the increase of the depth of the network. Its final model shown in Figure 3 uses 20 layers with small filters to obtain larger receptive field. Convergence speed is greatly affected by network depth. To get better performance and accelerate the speed at the same time, learning residuals has become a good choice, based on the fact that LR images and HR images share the same information to a large extent. The residuals between HR and LR images learned using extremely high learning rate combine LR images to generate final HR images. Note that images need bicubic interpolation to form input data, and all feature maps are in the same size by zero padding, so that the prediction effect of image edges is better. The parameters of VDSR are shown in Table 3. According to the experimental results, we find that 12 filters in convolution layer are enough to reconstruct space object images. Therefore, to train the model and reconstruct the images faster, we adjust the number of filters of convolution layer from 64 in [44] to 12 in this paper.

### 2.4. DRCN

**Figure 4:**
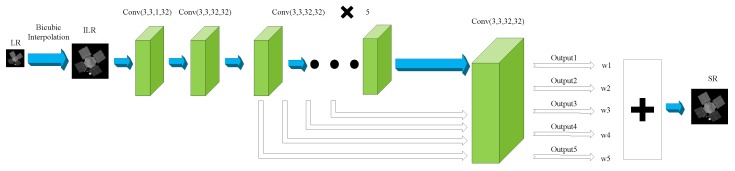
Network structure of DRCN used in this paper.

DRCN [43] (Deeply-Recursive Convolutional Networks) introduces a very deep recursive layer into the field of SR reconstruction. It may perform better if the depth of recursive layers increases, but the numbers of parameters do not increase much since all recursions share the same parameters which is contrary to convolution layers. It is also the obvious significance of importing recursive layers. The reconstruction results are obtained by weighted average of the results of each recursive convolution layer as shown in Figure 4. Bicubic interpolation is also a necessary procedure before training. The parameters of VDSR used in this paper are shown in Table 4. It should be noted that we changed the number of recursive layers from 16 in [43] into 5 for accelerating the training speed, because when the number of recursive layers is more than 5, the reconstructed results for space object images are almost invariant with the increase of recursive layers in our experiments.

## 3. Experiments and Analyses

### 3.1. Dataset

Our experiments use space object dataset BUAA-SID 1.0 [15,17] to explore the ability of the above four CNN-based SR methods in the application of space objects. BUAA-SID 1.0 cotains 20 categories of space objects, and each category has 230 images with the size of 240 × 320 forming a dataset with totally 4600 images. The images in each class are captured in different viewpoints.

We firstly divide all images in BUAA-SID 1.0 into 460 parts in order. For each part that contains ten images, nine images are selected randomly as training samples and one for testing or validation. In terms of the validation set, we randomly choose one image for every space object category, i.e., a total of 20 images. Thus the testing set contains 440 images. Since the images in BUAA-SID 1.0 have no background, we extract the region of interest (ROI) namely the external rectangle of the space object. Particularly, taking the probable impact of noise into account, we extract the external rectangle of all pixels whose gray value is above ten instead, and increase the length and width of the rectangle by 30 pixels without exceeding the image boundary. Since the four CNN-based SR models in Section 2 make no restrictions on the size of the input image, the image sizes in our dataset can be diverse. Therefore, to get more training data, every image in the training set is downsampled to 1, 0.95 and 0.9, generating 12,420 images at all. Furthermore, four patches are randomly extracted from every image as training HR patches, and 2, 3 and 4 times downsampling of these images are done to obtain corresponding LR patches. Therefore, the number of image pair in training set, validation set and testing set are 12,420, 20 and 440, respectively. It should be noted that the length and width of the external rectangle of extracted ROIs in testing set are 10 pixels larger than those in training set.

In addition, for better and comprehensive research and comparison, 91 images proposed in Yang et al. [46] which we name T91 are used as another independent training set, and two standard benchmark datasets, i.e., Set5 [47] and Set14 [48], are chosen for the corresponding testing set. We train the four popular CNN-based SR networks using T91 and BUAA-SID 1.0, respectively, and test the performance of them on three testing set when train on T91 dataset. By this way, we can not only compare our experimental results with the original paper to ensure its validity, but also explore the transfer performance of these networks between different data sets.

### 3.2. Index for Evaluation

We use peak signal-to-noise ratio (PSNR) and structural similarity index (SSIM) [49] as the metrics to evaluate the performance of each experiment.

Peak signal-to-noise ratio is widely used in the field of image quality assessment. It is defined by the maximum possible pixel value (denote as *L*) and the mean squared error (MSE) between images. Given the ground truth *X* with a total of *N* pixels and its corresponding constructed image $X_{SR}$, the MSE and the PSNR can be calculated by the following equations:

(1) $$MSE=\frac{1}{N}\sum_{i=1}^{N}{∥X\left(i\right)-X_{SR}\left(i\right)∥}_{2}$$

(2) $$PSNR=10\log_{10}\frac{L^{2}}{MSE}$$

The structural similarity index (SSIM) [49] focuses on measuring the structural similarity between images. It incorporates three relatively independent elements, including luminance, contrast and structure. The definition of SSIM is as follows:

(3) $$SSIM\left(X,X_{SR}\right)=\frac{\left(2\mu_{X}\mu_{X_{SR}}+C_{1}\right)\left(\sigma_{XX_{SR}}+C_{2}\right)}{\left({\mu_{X}}^{2}+{\mu_{X_{SR}}}^{2}+C_{1}\right)\left({\sigma_{X}}^{2}+{\sigma_{X_{SR}}}^{2}+C_{1}\right)}$$

where $C_{1}$ = ${\left(k_{1}L\right)}^{2}$ and $C_{2}$ = ${\left(k_{2}L\right)}^{2}$ are constants to avoid instability. The mean and the standard deviation of the ground truth *X* are denoted as $\mu_{X}$ and $\sigma_{X}$, respectively, and the mean and the standard deviation of constructed image $X_{SR}$ are denoted as $\mu_{X_{SR}}$ and $\sigma_{X_{SR}}$. $\sigma_{XX_{SR}}$ is the covariance between *X* and $X_{SR}$.

### 3.3. Training with Natural Images in Fixed Scale

We first train SRCNN, FSRCNN, VDSR and DRCN using T91 dataset to train three models for each network fixing the scale as 2, 3 and 4, respectively. Scale 2 means the spatial resolution of reconstructed image is twice of the input image. The larger the scale factor is, the worse the reconstructed image is, because the input image has relatively less information.

**Figure 5:**
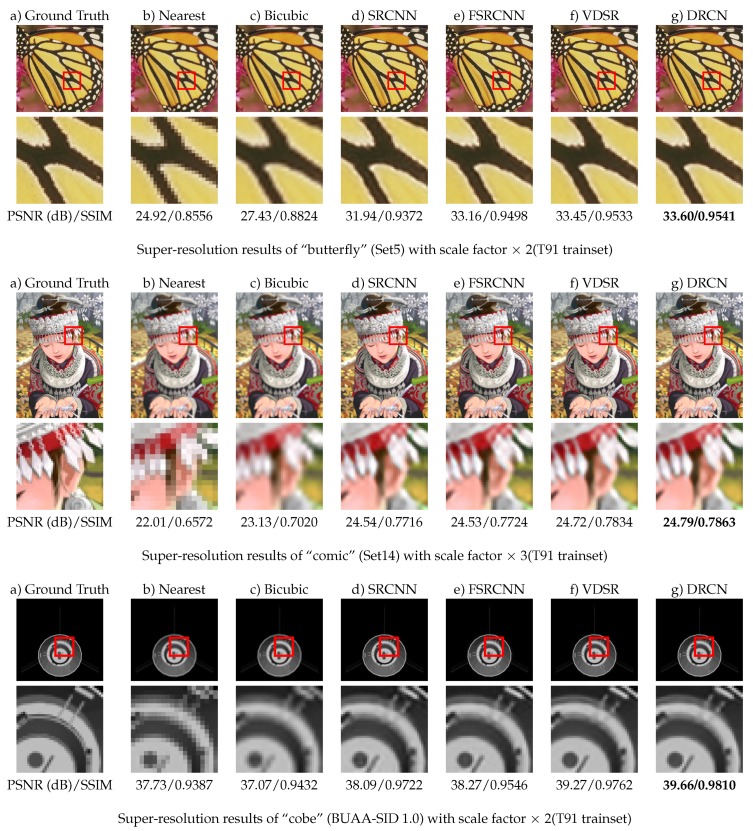
Visualization of super-resolution reconstruction.

The experimental results are shown in Table 5. The best results are marked in red and the second best in blue. The results show us that VDSR and DRCN perform better on natural images, while the reconstruction speed of FSRCNN is fastest except for the baseline bicubic method. When the testing data and training data is different to a large extent, DRCN and VDSR are also well adapted since they still rank the first and second, respectively. SRCNN, by contrast, do not work that well. FSRCNN works better than SRCNN, but worse than DRCN and VDSR. Figure 5 shows the visualization of sample reconstruction results on three testing sets.

In order to meet different requirements, we often need to train multiple networks according to the reconstruction scale, because the network trained by fixing the scale as a certain number is only adapted to reconstruct this certain scale, i.e., fixed scale super-resolution. When the testing scale is different from the training scale, the reconstruction result will be worse. In addition, training several networks means multiplied number of parameters and time consuming process of training. This is a problem that cannot be ignored in practical application.

### 3.4. Training with Natural Images in Multiple Scales

In response to the problem mentioned in Section 3.3, we use hybrid training strategy. That is to say we train a single model that is universal to different reconstruction scales by randomly selecting HR/LR image patches of all scales as input data. In this way, the parameters to be trained are greatly reduced. Images can be reconstructed at any scale using one set of model parameters, i.e., multiple scale super-resolution.

Because of the existence of deconvolution layer in FSRCNN, the structure of network will be different if the training HR/LR patches are in different scales. So FSRCNN cannot be trained to reconstruct different scale images using this strategy. Multiple scale super-resolution results of the other three networks trained on T91 dataset are shown in Table 6. PSNR- and SSIM- denote the difference between the multiple scale experimental results and fixed scale super-resolution reconstruction results.

The experimental results prove that it is feasible to reconstruct the image at any scale by using this training strategy. The performance of VDSR and DRCN is relatively good. Compared with the fixed scale super-resolution results in Table 5, multiple scale super-resolution results are not much different. The strategy of mixing HR/LR patches of different scales as training set overcomes the shortcoming that a new requirement of a certain scale SR needs a new model. It may greatly improve the efficiency of reconstruction.

### 3.5. Training with Space Object Images

#### 3.5.1. Comparison of Fixed Scale and Multiple Scale

**Figure 6:**
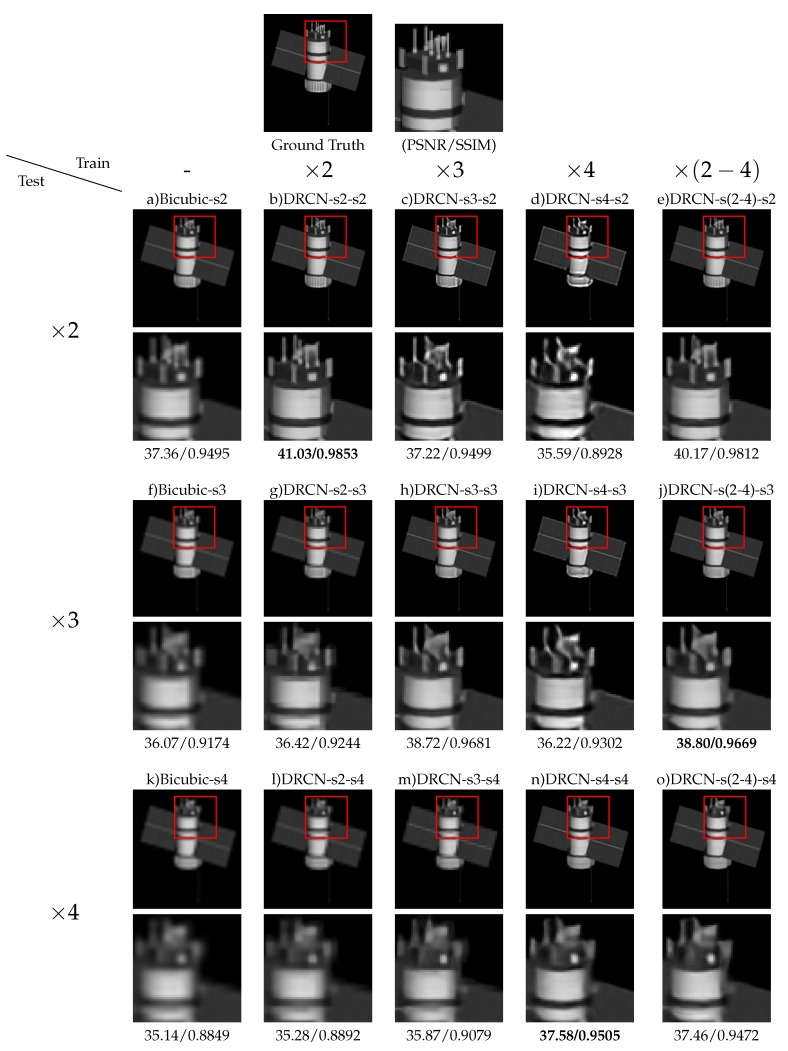
Scale factor experiment for “glonas” in BUAA-SID 1.0. The method sm−sn means the method is trained for ×m SR and tested for ×n SR.

For further comparison and analysis in the field of space objects, we perform more comprehensive experiments using BUAA-SID 1.0 dataset. We design experiments for each network to explore the performance of models trained by fixing scale or mixing scales when testing at a certain scale. That is to say, we test the reconstruction ability at three scales of every model we trained, not just the scale it is trained for. The experimental results of SRCNN, VDSR and DRCN are shown in Table 7, Table 8 and Table 9, respectively. Figure 6 shows the results of the comparison. In addition, in order to ensure the results are statistically significant, we train 3 different models repeatedly for every experiments, and report the means and standard deviations of PSNR and SSIM for evaluation.

By analyzing the experimental results of the above three methods trained by multiple scale and single scale image pairs, we can get a consistent conclusion. Using multiple scale image pairs to train the network can achieve the purpose of reconstructing HR images at any scale and the model performs well. It is only a little worse than the model whose training and testing scale is perfectly matched. While models trained by fixed scale cannot fit well when the testing scale does not match the training scale. Besides, the bigger the gap between them is, the worse the effect appears. In practical application, it is often necessary to reconstruct the space object image at any scale, but not just a fixed scale. Therefore, getting a single model which is universal to reconstruct HR images at any scale is a better choice. As for the performance of each individual network, it can be can easily see in Table 10 that DRCN is the best, VDSR is the second and SRCNN is the worst.

Figure 6 shows scale factor experiment for “glonas” in BUAA-SID 1.0. It can explain the experiment results and conclusion mentioned above more clearly. The method sm−sn means the method is trained for scale ×m SR and tested for scale ×n SR. We can observe that if the scale of training does not include the scale of testing, the reconstructed image has poor image quality. Specifically, if the scale of testing bigger than the scale of training, i.e., ($s_{test}$ > $s_{train}$), the SR results are blurry and the the high frequency textures are significantly lost. In construct, if $s_{test}$ < $s_{train}$, the SR results show unnatural artifacts caused by over-enhancing high-frequency edges. In addition, if the network is trained by multiple scale, the reconstructed images for any scale have satisfying quality.

#### 3.5.2. Comparison of Direct Training and Transfer Training

How to train our networks is also an important factor that may affect the final results. Direct training and transfer training are two common choices. Direct training means training a randomly initialized network directly using space object training set, while transfer training in our experiments is pre-training the network parameters firstly with T91 training set, and then using space object data to fine-tune the pre-trained network. We compare the effect of these two training methods on the task of reconstruct HR images of space objects.

**Figure 7:**
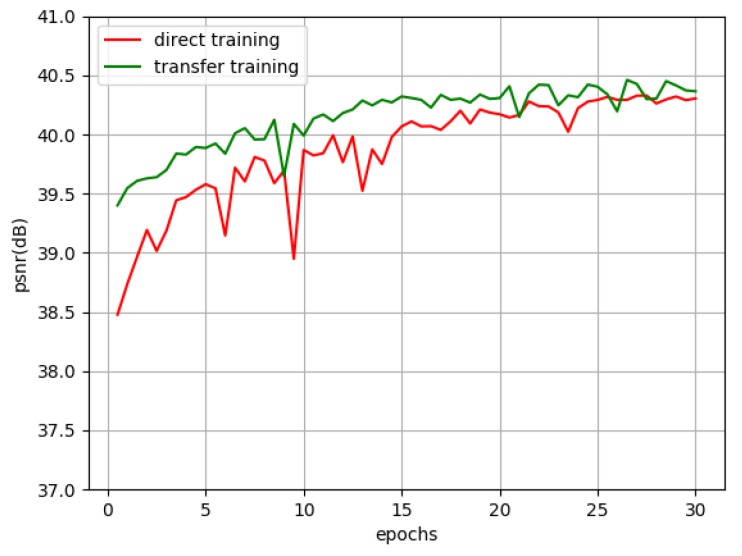
Performance of DRCN training by different methods.

We can see the final results of four networks in Table 11. There is a little difference between direct training and transfer training, and the results of transfer training is slightly better than that of direct training. This is to say transfer training cannot obviously improve the reconstruction effect of network on space object dataset. However, it can be seen from the training process in Figure 7 that transfer training can converge faster. The results indicate that transfer training is beneficial for accelerating network convergence, and the features learned by natural images (T91 training set) are helpful to super-resolution of space objects images.

**Figure 8:**
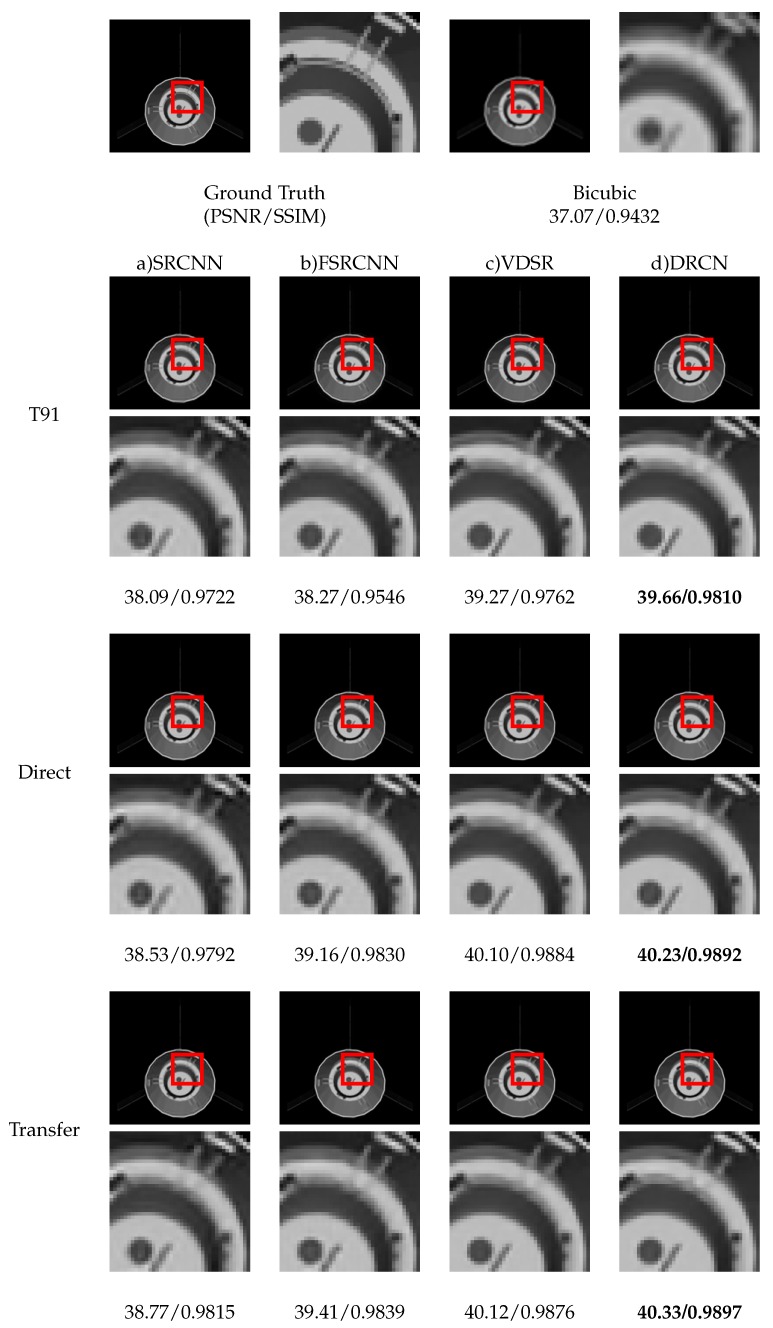
Super-resolution results of “cobe” (BUAA-SID 1.0) with scale factor × 2. Models are trained on T91, directly trained on BUAA-SID 1.0, and transfer trained from T91 respectively.

Figure 8 shows the reconstruction results of different training methods. Notice that whether it is direct training or transfer training, the testing results are better than that trained by natural image dataset. This illustrates that it is necessary and effective to use the same or similar images with the image category to be reconstructed as the training set.

#### 3.5.3. Computational Complexity

The computational complexity of the methods is also an important factor to measure their time efficiency and memory cost. We compare the times of multiplication calculation and the number of parameters of the four CNN-based networks, in order to theoretically analyze their computational complexity. Results in Table 12 show that FSRCNN has the least theoretical calculations and parameters, thus, it will run faster and cost less memory. Table 5 also validates that FSRCNN costs the least running time when reconstructing images. The only inconsistency between Table 5 and Table 12 is VDSR. VDSR runs slowest while its theoretical computational complexity is the second best. This may be caused by the GPU acceleration when implementing the CNN-based networks. Since all of the networks using GPU for accelerating, the actual reconstruction time is not completely linear correlated with the theoretical calculations. In Table 5, the running speeds of SRCNN, VDSR and DRCN are not significantly different. This inspires us to implement CNN-based SR networks on a programming platform with better hardware acceleration for CNN.

#### 3.5.4. Noise Robustness

**Figure 9:**
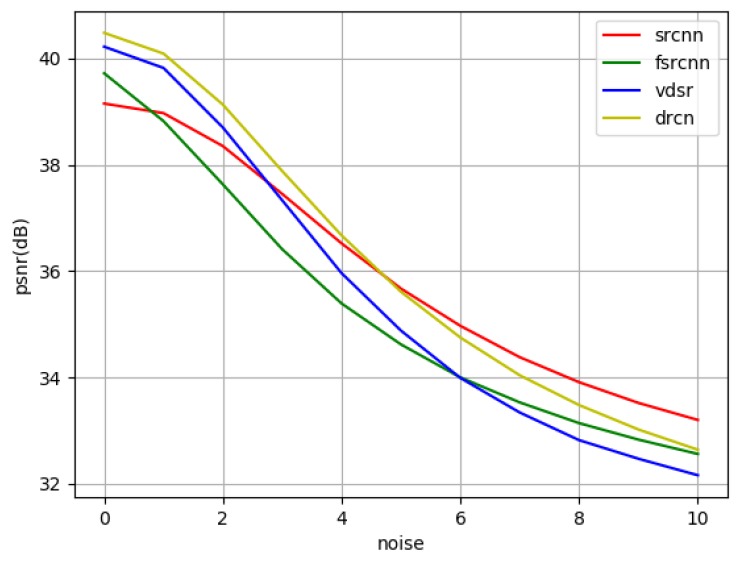
PSNR curve with different std of Gaussian noise.

In practice, the space object images to be reconstructed may have different levels of noise, and the addition of noise will have a certain impact on the reconstruction effect. So it is necessary to experimentally test the anti-noise performance of the four CNN-based networks. Gaussian noise with a standard deviation (std) of 1–10 is added to the LR images of the testing set, as well as salt and pepper noise and Poisson noise. The super-resolution reconstruction results are compared with the noise-free HR image to obtain the PSNR/SSIM between the them. Table 13 shows the detail results and Figure 9 makes it easier to compare and analyze.

We can see from Table 13 and Figure 9 that the reconstruction effect of these four networks is affected to some extent with the increase of noise, among which the SRCNN is less affected by noise than the other three networks. In our experiments, we use a noise-free training set to train the SR networks, therefore the well-trained networks may not study suitable strategy to process images with various modes of noise. Generally, SRCNN has better noise robustness than other three network. The reason may be that SRCNN has the simplest structure, and thus, the model is less affected by noise. This indicates that the SR reconstruction algorithms based on deep neural networks may not have good anti-noise ability when training with noise free data, and the addition of noise has a great impact on their performance. Noise robustness may be a new branch of CNN-based SR reconstruction that need to be studied and improved.

## 4. Discussion

The analysis of the advantages and disadvantages of these four deep learning models can help choose the most suitable model for single image super-resolution of space objects.

In the circumstance that we do not have enough space object images to train a deep learning model, we take a model trained by natural images as shown in Section 3.3 instead. We can see from Table 5 that FSRCNN runs fastest to reconstruct HR images than other three models. In terms of reconstructed quality, DRCN and VDSR are the first and second, respectively. SRCNN does not work so well. FSRCNN works better than SRCNN, but worse than DRCN and VDSR. If we want to use a single model trained by natural images to reconstruct multiple scales, DRCN is the best model that is more generalized to space object images.

Mostly previous work using single scale LR/HR trainset to train the network. According to Table 7, Table 8 and Table 9, the network trained by fixing the scale as a certain number is only adapted to reconstruct this certain scale. The network performs poorly when the testing scale does not meet the training scale. Such a shortcoming limits the application of super-resolution for space object images. In order to overcome the weakness, we use hybrid training strategy. The experimental results in Table 7, Table 8 and Table 9 show that multiple scale network can achieve comparable results against fixed scale ones, especially when the testing scale is high (3, 4 in our experiments). It proves that it is feasible to reconstruct the image at any scale by using this training strategy. In addition, VDSR and DRCN are more suitable to use the strategy because their networks are complicated enough to process different scales images. Therefore, hybrid training strategy is meaningful for super-resolution of space object images. The well-trained network can process input images of all scales, i.e., the network can reconstruct the input image to any size, and the results are much better than the images generated by interpolation method, e.g., bicubic.

We also design the experiments about direct learning and transfer learning in Section 3.5.2. The results of transfer training is slightly better than that of direct training. Figure 7 shows that transfer training can converge faster. This indicates that transfer training is beneficial for accelerating network convergence and improving reconstructed results.

Furthermore, we analyze the computational complexity of these four deep learning models. According to Table 12, FSRCNN takes the lowest the least theoretical computational complexity. However, in order to get better efficiency and lower memory cost in practice, we should also consider the software optimization and hardware acceleration when implementing CNN-based SR models on a programming platform.

At last, we analyze noise robustness of four networks. All the four methods trained by noise free data cannot process images with noise effectively. Generally, SRCNN has better noise robustness than the other three network. If the image to be reconstructed contains strong noise, a feasible approach is to first denoise the image and then construct it.

Overall, SRCNN has the simplest structure, but the main body and edge of the space target are not well reconstructed by SRCNN since only three layers of SRCNN limit its ability to express and reconstruct space target image features. FSRCNN contains eight layers and uses a deconvolution layer to raise image resolution, because the first seven convolutional layers are calculated on low resolution images, FSRCNN runs faster than SRCNN while its SR performance is unremarkable. VDSR reconstructs the residual image, making it easier to study the difference between LR and HR. The edge and texture of the space target reconstructed by VDSR are clearer. DRCN uses recursive convolution networks. Its output layer takes the advantages of the information of the 3rd to 7th layers, thus, the main structure and edge details of the space target can be super-resolved best among the four CNN-based models, in both fixed scale and multiple scales. As a result, we suggest using DRCN fine-tuned from pretrained model on natural dataset as CNN-based SR model for space-based imaging sensors.

## 5. Conclusions

To meet the needs of image super-resolution in space applications, we have comparably studied four recent popular models for single image super-resolution based on convolutional neural networks. We not only explore the difference in the performance of these models, but also find some common properties which may be more important to inspire further research. Firstly, a multiple scale training strategy has been proven as an efficient way to obtain a single model to reconstruct HR images at any scale. Solving multiple scale SR tasks with one model is more valuable in practice. Secondly, transfer training makes the network easier to converge, and has slightly better results than training the initialized network using space object data directly. Thirdly, testing results will be better if the consistency between the training set and testing set is high. It is the key to success on a particular mission, but it is also an obstacle to expansion on other tasks. Finally, noise is a killer for image super-resolution because it is also amplified during reconstruction. In general, DRCN is the best model of the four models in this paper, since DRCN performs best in super-resolution of space object images in fixed scale and multiple scale. According to this work, researchers may see the advantages and disadvantages of CNN-based super-resolution methods more clearly and then promote the development of image super-resolution in space applications.

## Figures and Tables

**Table 1:** Parameters of SRCNN used in this paper.

**Input**	Bicubic interpolation of LR images
**Number of layers**	3
**Residual unit**	No
**Parameters of 1st layer**	9×9×1×64
**Parameters of 2nd layer**	5×5×64×32
**Parameters of 3rd layer**	5×5×32×1
**Learning rate**	$1\times 10^{-4}$

**Table 2:** Parameters of FSRCNN used in this paper.

**Input**	LR images
**Number of layers**	8
**Residual unit**	No
**Parameters of 1st layer**	5×5×1×56
**Parameters of 2nd layer**	1×1×56×12
**Parameters of 3rd-6th layer**	3×3×12×12
**Parameters of 7th layer**	1×1×12×56
**Parameters of 8th layer**	9×9×56×1
**Learning rate**	$1\times 10^{-3}$

**Table 3:** Parameters of VDSR used in this paper.

**Input**	Bicubic interpolation of LR images
**Number of layers**	20
**Residual unit**	Yes
**Parameters of 1st layer**	3×3×1×12
**Parameters of 2nd-19th layer**	3×3×12×12
**Parameters of 20th layer**	3×3×12×1
**Learning rate**	$1\times 10^{-3}$

**Table 4:** Parameters of DRCN used in this paper.

**Input**	Bicubic interpolation of LR images
**Number of layers**	9
**Residual unit**	No
**Parameters of 1st layer**	3×3×1×32
**Parameters of 2nd layer**	3×3×32×32
**Parameters of 3rd-7th layer**	3×3×32×32
**Parameters of 8th layer**	3×3×32×32
**Parameters of 9th layer**	3×3×32×1
**Learning rate**	$1\times 10^{-3}$

**Table 5:** Fixed scale super-resolution results of networks trained on T91 dataset. The  red font indicates the best performance, while the blue font indicates the second best.

Methods	Scale	Set5 PSNR/SSIM/TIME(s)	Set14 PSNR/SSIM/TIME(s)	BUAA-SID1.0 PSNR/SSIM/TIME(s)
	2	33.73/0.9233/0.001	30.29/0.8704/0.001	36.99/0.9374/0.001
Bicubic	3	30.53/0.8685/0.001	27.73/0.7965/0.001	35.63/0.8877/0.001
	4	28.61/0.8250/0.001	26.27/0.7474/0.001	34.80/0.8444/0.001
	2	36.49/0.9469/0.341	32.28/0.9010/0.317	38.77/0.9640/0.162
SRCNN	3	32.76/0.9038/0.342	29.30/0.8301/0.336	36.94/0.9279/0.170
	4	30.42/0.8617/0.340	27.53/0.7784/0.328	35.77/0.8878/0.166
	2	36.95/0.9512/0.267	32.55/0.9049/0.256	38.92/0.9535/0.125
FSRCNN	3	32.75/0.9043/0.266	29.29/0.8301/0.271	36.56/0.8878/0.128
	4	30.56/0.8642/0.273	27.58/0.7795/0.268	35.49/0.8512/0.119
	2	37.02/0.9514/0.371	32.59/0.9053/0.376	39.30/0.9651/0.188
VDSR	3	33.11/0.9098/0.368	29.50/0.8345/0.384	37.15/0.9257/0.196
	4	30.75/0.8712/0.372	27.72/0.7845/0.383	35.94/0.8861/0.177
	2	37.23/0.9522/0.330	32.74/0.9061/0.360	39.57/0.9711/0.181
DRCN	3	33.18/0.9107/0.331	29.55/0.8356/0.366	37.36/0.9327/0.175
	4	30.86/0.8727/0.319	27.79/0.7867/0.363	36.17/0.8968/0.186

**Table 6:** Multiple scale super-resolution results of networks trained on T91 dataset. The  red font indicates the best performance, while the blue font indicates the second best.

Test Data	Scale	SRCNN PSNR/SSIM/PSNR-/SSIM-	VDSR PSNR/SSIM/PSNR-/SSIM-	DRCN PSNR/SSIM/PSNR-/SSIM-
	2	34.17/0.9283/−2.32/−0.0186	36.61/0.9490/−0.41/−0.0024	36.59/0.9481/−0.64/0.0041
Set5	3	31.73/0.8894/−1.03/−0.0144	33.02/0.9087/−0.09/−0.0011	32.98/0.9082/−0.20/−0.0025
	4	29.64/0.8482/−0.78/−0.0135	30.77/0.8708/+0.02/−0.0004	30.69/0.8699/−0.17/−0.0028
	2	30.98/0.8837/−1.30/−0.0173	32.33/0.9025/−0.26/−0.0028	32.29/0.9018/−0.45/−0.0043
Set14	3	28.64/0.8164/−0.66/−0.0137	29.41/0.8331/−0.09/−0.0014	29.40/0.8329/−0.40/−0.0027
	4	26.95/0.7655/−0.58/−0.0129	27.71/0.7845/−0.01/0.0000	27.68/0.7838/−0.11/−0.0029
	2	37.42/0.9511/−1.35/−0.0129	38.78/0.9622/ −0.52/−0.0029	38.88/0.9651/ −0.69/−0.0060
BUAA-SID1.0	3	36.37/0.9159/−0.57/−0.0120	37.00/0.9263/ −0.15/+0.0006	37.14/0.9317/ −0.22/−0.0010
	4	35.49/0.8782/−0.28/−0.0096	35.97/0.8881/ +0.03/+0.0020	35.99/0.8941/ −0.18/−0.0027

**Table 7:** Cross-scale experiments of SRCNN trained and tested on BUAA-SID 1.0 (mean ± standard deviation). The  red font indicates the best performance, while the blue font indicates the second best.

Index	Scale	Bicubic	SRCNN × 2	SRCNN × 3	SRCNN × 4	SRCNN × 2,3,4
	2	36.99	39.05 ± 0.09	36.04 ± 0.03	34.85 ± 0.08	38.26 ± 0.03
**PSNR**	3	35.63	36.02 ± 0.02	37.24 ± 0.03	35.51 ± 0.12	37.00 ± 0.04
	4	34.80	34.95 ± 0.01	35.35 ± 0.02	36.16 ± 0.04	36.15 ± 0.06
	2	0.9374	0.9700 ± 0.0007	0.9120 ± 0.0007	0.8206 ± 0.0018	0.9633 ± 0.0002
**SSIM**	3	0.8877	0.8986 ± 0.0002	0.9377 ± 0.0006	0.8848 ± 0.0015	0.9330 ± 0.0010
	4	0.8444	0.8523 ± 0.0002	0.8716 ± 0.0007	0.9064 ± 0.0009	0.9042 ± 0.0015

**Table 8:** Cross-scale experiments of VDSR trained and tested on BUAA-SID 1.0 (mean ± standard deviation). The  red font indicates the best performance, while the blue font indicates the second best.

Index	Scale	Bicubic	VDSR × 2	VDSR × 3	VDSR × 4	VDSR × 2,3,4
	2	36.99	40.21 ± 0.07	36.52 ± 0.15	35.35 ± 0.05	39.45 ± 0.04
**PSNR**	3	35.63	35.95 ± 0.01	37.82 ± 0.03	35.95 ± 0.05	37.69 ± 0.02
	4	34.80	34.98 ± 0.04	35.29 ± 0.02	36.62 ± 0.01	36.61 ± 0.03
	2	0.9374	0.9781 ± 0.0004	0.9309 ± 0.0029	0.8848 ± 0.0026	0.9724 ± 0.0004
**SSIM**	3	0.8877	0.8945 ± 0.0002	0.9470 ± 0.0002	0.9084 ± 0.0021	0.9430 ± 0.0007
	4	0.8444	0.8509 ± 0.0001	0.8642 ± 0.0008	0.9164 ± 0.0005	0.9139 ± 0.0011

**Table 9:** Cross-scale experiments of DRCN trained and tested on BUAA-SID 1.0 (mean ± standard deviation). The  red font indicates the best performance, while the blue font indicates the second best.

Index	Scale	Bicubic	DRCN × 2	DRCN × 3	DRCN × 4	DRCN × 2,3,4
	2	36.99	40.48 ± 0.03	36.52 ± 0.01	35.15 ± 0.08	39.75 ± 0.09
**PSNR**	3	35.63	35.98 ± 0.02	38.00 ± 0.02	36.05 ± 0.08	37.86 ± 0.06
	4	34.80	34.98 ± 0.01	35.37 ± 0.01	36.79 ± 0.01	36.61 ± 0.03
	2	0.9374	0.9798 ± 0.0001	0.9287 ± 0.0007	0.8554 ± 0.0034	0.9753 ± 0.0006
**SSIM**	3	0.8877	0.8955 ± 0.0006	0.9515 ± 0.0001	0.9054 ± 0.0015	0.9487 ± 0.0012
	4	0.8444	0.8520 ± 0.0002	0.8677 ± 0.0006	0.9164 ± 0.0005	0.9199 ± 0.0016

**Table 10:** Multiple scale super-resolution results of networks trained and tested on BUAA-SID 1.0 (mean ± standard deviation). The  red font indicates the best performance, while the blue font indicates the second best.

Scale	Bicubic PSNR/SSIM	SRCNN × 2,3,4 PSNR/SSIM	VDSR × 2,3,4 PSNR/SSIM	DRCN × 2,3,4 PSNR/SSIM
2	36.99/0.9374	38.26 ± 0.03/0.9633 ± 0.0002	39.45 ± 0.04/0.9724 ± 0.0004	39.75 ± 0.09/0.9753 ± 0.0006
3	35.63/0.8877	37.00 ± 0.04/0.9330 ± 0.0010	37.69 ± 0.02/0.9430 ± 0.0007	37.86 ± 0.06/0.9477 ± 0.0012
4	34.95/0.8521	36.15 ± 0.06/0.9042 ± 0.0015	36.61 ± 0.03/0.9139 ± 0.0011	36.74 ± 0.05/0.9199 ± 0.0016

**Table 11:** Comparison of different training methods.

Test Data	Training Method	Scale	SRCNN PSNR/SSIM	FSRCNN PSNR/SSIM	VDSR PSNR/SSIM	DRCN PSNR/SSIM
BUAA-	direct training	2	39.15/0.9709	39.72/0.9743	40.22/0.9786	40.48/0.9798
SID1.0	transfer training	2	39.41/0.9731	39.88/0.9745	40.25/0.9789	40.58/0.9804

**Table 12:** The comparison of computational complexity for an input image of size m×n. The  red font indicates the best performance, while the blue font indicates the second best.

Term	Scale	SRCNN	FSRCNN	VDSR	DRCN
	2	$2.29\times 10^{5}m\times n$	$2.61\times 10^{4}m\times n$	$9.42\times 10^{4}m\times n$	$2.60\times 10^{5}m\times n$
Multiplication times	3	$5.15\times 10^{5}m\times n$	$4.86\times 10^{4}m\times n$	$2.12\times 10^{5}m\times n$	$5.86\times 10^{5}m\times n$
	4	$9.19\times 10^{5}m\times n$	$8.05\times 10^{4}m\times n$	$3.77\times 10^{5}m\times n$	$1.04\times 10^{6}m\times n$
	2	$5.73\times 10^{4}$	$1.26\times 10^{4}$	$2.38\times 10^{4}$	$6.53\times 10^{4}$
Number of parameters	3	$5.73\times 10^{4}$	$1.26\times 10^{4}$	$2.38\times 10^{4}$	$6.53\times 10^{4}$
	4	$5.73\times 10^{4}$	$1.26\times 10^{4}$	$2.38\times 10^{4}$	$6.53\times 10^{4}$

**Table 13:** The effect of noise on reconstruction results. The  red font indicates the best performance, while the blue font indicates the second best.

Noise Type	SRCNN PSNR/SSIM	FSRCNN PSNR/SSIM	VDSR PSNR/SSIM	DRCN PSNR/SSIM
None	39.15/0.9709	39.72/0.9744	40.22/0.9786	40.48/0.9798
Gaussian (std = 1)	38.97/0.9672	38.82/0.9262	39.82/0.9652	40.09/0.9746
Gaussian (std = 2)	38.35/0.9450	37.63/0.8645	38.70/0.9119	39.13/ 0.9353
Gaussian (std = 3)	37.45/ 0.9073	36.41/0.8022	37.33/0.8638	37.88/ 0.8724
Gaussian (std = 4)	36.52/ 0.8636	35.39/0.7442	35.96/0.7592	36.67/ 0.8087
Gaussian (std = 5)	35.67/0.8179	34.62/0.6941	34.88/0.6873	35.61/0.7509
Gaussian (std = 6)	34.97/0.7741	34.00/0.6488	33.99/0.6246	34.75/0.7000
Gaussian (std = 7)	34.38/0.7322	33.53/0.6088	33.34/0.5699	34.04/0.6549
Gaussian (std = 8)	33.91/0.6938	33.14/0.5736	32.82/0.5229	33.48/0.6146
Gaussian (std = 9)	33.52/0.6577	32.83/0.5429	32.47/0.4822	33.02/0.5785
Gaussian (std = 10)	33.20/0.6248	32.56/0.5138	32.16/0.4466	32.64/0.5462
Salt and pepper (0.02)	33.96/0.7473	33.55/0.6743	35.04/ 0.7271	34.33/0.6770
Poisson	35.35/0.8861	35.36/0.8844	35.49/0.8888	35.71/0.9001
